# Forging out-of-equilibrium supramolecular gels

**DOI:** 10.1038/s44160-024-00623-4

**Published:** 2024-09-06

**Authors:** Simona Bianco, Fin Hallam Stewart, Santanu Panja, Asra Zyar, Emma Bowley, Marko Bek, Roland Kádár, Ann Terry, Roberto Appio, Tomás S. Plivelic, Mahon Maguire, Harish Poptani, Marco Marcello, Ravi R. Sonani, Edward H. Egelman, Dave J. Adams

**Affiliations:** 1 School of Chemistry, University of Glasgow, Glasgow, UK; 2 Department of Industrial and Materials Science, Chalmers University of Technology, Göteborg, Sweden; 3 MAX IV Laboratory, Lund University, Lund, Sweden; 4 Centre for Preclinical Imaging, Department of Molecular and Clinical Cancer Medicine, University of Liverpool, Liverpool, UK; 5 Centre for Cell Imaging, University of Liverpool, Liverpool, UK; 6 Department of Biochemistry and Molecular Genetics, University of Virginia, Charlottesville, VA USA

**Keywords:** Soft materials, Biomaterials, Supramolecular polymers, Self-assembly

## Abstract

The design of supramolecular hydrogels comprising aligned domains is important for the fabrication of biomimetic materials and applications in optoelectronics. One way to access such materials is by the self-assembly of small molecules into long fibres, which can be aligned using an external stimulus. Out-of-equilibrium supramolecular gels can also be designed, where pre-programmed changes of state can be induced by the addition of chemical fuels. Here we exploit these dynamic properties to form materials with aligned domains through a ‘forging’ approach: an external force is used to rearrange the underlying network from random to aligned fibres as the system undergoes a pre-programmed gel-to-sol-to-gel transition. We show that we can predictably organize the supramolecular fibres, leading to controllable formation of materials with aligned domains through a high degree of temporal control.

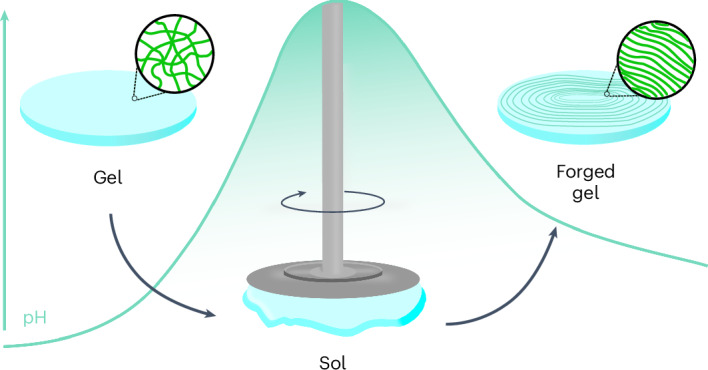

## Abstract (web summary)

Dynamic supramolecular systems can be designed to adapt phases in a pre-programmable way. Here the transient nature of a gel system is exploited, in combination with the application of mechanical stimuli, to obtain soft materials with aligned fibres in a controllable way.

## Main

Transient, dynamic and dissipative supramolecular systems have recently attracted substantial interest^1–9^. In such systems, supramolecular structures form and change in a pre-programmed manner, controlled by external stimuli such as chemical or light triggers, and typically result in changes in molecular structure^10–12^. These transient and dynamic systems have been investigated in the bulk, as well as within droplets^13,14^. The processes occurring in these systems are often comparable or analogous to those found within cells, especially when confined within droplets. For example, van Esch’s group have shown how a gel with a pre-programmed lifetime can be formed by the addition of a chemical fuel^15^. Addition of the fuel results in a chemical reaction, leading to the formation of a low-molecular-weight gelator, a molecule that self-assembles into supramolecular fibres to immobilize the solvent. Once the fuel has been used up, a second chemical reaction dominates, leading to the molecule being converted back to the starting compound which can no longer act as a gelator. Overall, this process leads to a sol-to-gel-to-sol transition with controllable gel lifetimes. There are many examples now of similar processes resulting in changes of state based on a range of different reactions, as well as the use of light or electrochemical stimuli to modify molecular structure and hence alter the aggregation type^16–28^.

However, almost all the reported systems operate within a static environment, in which external conditions are typically kept constant while the chemical processes are ongoing. There is no reason why this needs to be the case. External forces could be applied to manipulate these systems as they are evolving chemically, and it is possible to imagine how the supramolecular structures formed at different times could be affected by an external force to a greater or lesser extent. In particular, mechanical forces can be used as directional stimuli to impart anisotropy in supramolecular gels^29–32^, and shaking has been used to mix components and lead to changes in dynamic systems in a closed system^33^. Long-range alignment is often found within biological systems, and the design of soft materials comprising of aligned fibrils is attractive for tissue engineering applications^34^. For this purpose, extrusion techniques have been used to achieve aligned nanomaterials^32,35^, and xerogels containing aligned fibres have been formed under shear^36^. It is worth noting that a range of forces have been used to affect supramolecular systems including ultrasound^37,38^, sound waves^39–41^ and magnetic fields^42–47^.

We have previously described systems undergoing pre-programmed gel-to-sol-to-gel transitions by incorporating two competing pH triggers, resulting in a pH increase and subsequent decrease to anneal a supramolecular gel^48,49^. Following initial gelation at low pH, a conversion to a free-flowing micellar state is achieved at high pH before a gel is reformed as the pH gradually decreases. Critically, the final gel exhibits improved mechanical properties underpinned by a change in the self-assembled structures driven by the pH cycle.

**Fig. 1:**
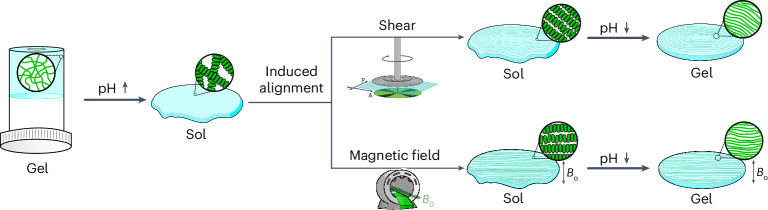
Forging supramolecular gels using external stimuli to induce alignment. A cartoon of the work described in this Article: alignment is induced in a system undergoing a chemically triggered gel-to-sol-to-gel cycle by applying external invasive (shear) and non-invasive (magnetic field) stimuli to impart changes in the organization of the self-assembled fibres.

In this Article, we describe what we call a ‘forging’ approach, inspired by how blacksmiths work heated metal into desired shapes. We report supramolecular systems that change with time in a pre-programmed manner while simultaneously an external force is applied; this contrasts with previous examples where the system is quiescent (Fig. 1). Owing to the pre-programmable nature of the system, the force can be applied with a high level of temporal control. We show how the application of the force results in changes to the mesoscale organization of the supramolecular structures present, leading to the formation of aligned fibrillar domains while also changing the mechanical properties of the resulting gel. We further show how anisotropy in these systems can be achieved non-invasively by evolution of these materials within a strong magnetic field.

## Results and discussion

**Fig. 2:**
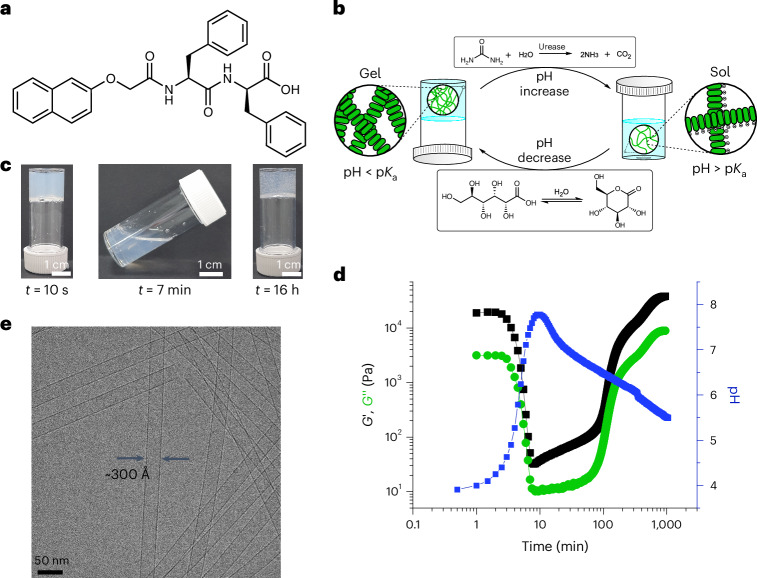
A pre-programmed gel-to-sol-to-gel system. **a**, The chemical structure of l,d-2NapFF. **b**, A cartoon of the gel-to-sol-to-gel process. **c**, Photographs of the system at 10 s, 7 min and 16 h (scale bar, 1 cm). **d**, The change in rheology and pH with time for a system containing l,d-2NapFF in the presence of urea, urease and GdL. The lines and circles are colour coded to match the coloured axis titles. **e**, Cryo-EM of l,d-2NapFF (scale bar, 50 nm). In **c** and **d**, [l,d-2NapFF] = 5 mg ml^−1^, [urea] = 0.04 M, [urease] = 0.4 mg ml^−1^ and [GdL] = 14.3 mg ml^−1^. Source data

We focus on a transient gel-to-sol-gel system. l,d-2NapFF (Fig. 2a) is an effective low-molecular-weight gelator^50^. On dilution of a high concentration solution of l,d-2NapFF in dimethyl sulfoxide (DMSO) with water to give a final water:DMSO ratio of 90:10, a gel is formed at a final concentration of l,d-2NapFF of 5 mg ml^−1^ at a pH of 4.0. At this point, the gels are formed by spherulitic domains of supramolecular fibres that jam together^48^. Such solvent-triggered gels are stable for extended periods of time. Small angle X-ray scattering (SAXS) shows that the structures underpinning the gel network are thin-walled nanotubes with a radius of 14.7 nm and a wall thickness of 3.4 nm (Supplementary Fig. 11 and Supplementary Table 3).

To induce a pre-programmed phase transition, dilution can be carried out with an aqueous solution of urease, urea and glucono-δ-lactone (GdL) instead of pure water. Initially, a solvent-triggered gel is formed at low pH (~4). As the pH increases, driven by the production of ammonia through the reaction between urea and urease, the gel converts to a micellar solution of nanotubes due to the deprotonation of the terminal carboxylic acid^18,51,52^. At high pH, the nanotubes will be charged and hence direct interactions will be unlikely. When the pH reaches a sufficiently high value, the hydrolysis of GdL to gluconic acid becomes predominant, leading to a slow decrease in pH and re-gelation once the pH drops below the apparent p*K*_a_ of the gelator (~6.0) (ref. ^50^). Hence, overall we have a gel-to-sol-to-gel transition (Fig. 2b), with the timescales of each stage being determined by the concentrations of urease, urea and GdL used. While direct addition of acid and bases can be used to achieve such changes, irreproducible and inhomogeneous systems are often obtained in this way owing to fast mixing^53^. Further, using a pre-programmable pH cycle allows highly reproducible temporal control over the phase changes.

This process can be followed by eye (Fig. 2c) and by rheology (Fig. 2d and Supplementary Fig. 2a). Initially, a gel is formed as shown by the storage modulus (*G*′) being an order of magnitude higher than the loss modulus (*G*″). As the pH increases, both *G*′ and *G*″ decrease, showing that a micellar solution is formed. This is further supported by the observation of a *G*′ and *G*′′ cross-over when the data are collected with a frequency of 50 rad s^−1^ (Supplementary Fig. 2a), in line with our previous data^54^. Once the hydrolysis of GdL becomes more dominant and the pH decreases, gelation reoccurs, represented by an increase of both rheological moduli. As we have shown for related systems^48^, this can be thought of as a pH-induced ‘annealing’ process, which often results in improvement of the final mechanical properties of the material compared with the original system.

The solution phase obtained at high pH contains micellar aggregates formed by l,d-2NapFF with a deprotonated terminal carboxylic acid. In this phase, l,d-2NapFF forms well-defined nanotubes at high pH as shown by SAXS^50^ and cryo-EM (Fig. 2e and Supplementary Fig. 1)^55^ that exhibit shear-thinning behaviour under shear (Supplementary Fig. 3b). Here, the nanotube formation is primarily driven by π–π stacking between the aromatic rings of the l,d-2NapFF molecules, which then twist in a left-handed helical manner to form the large, hollow structures^55^. Interestingly, related nanotubes can be aligned using shear or in a magnetic field^42,56^. Alignment under shear is expected for long anisotropic structures^36,57^. Alignment in a magnetic field is possible owing to high diamagnetic anisotropy resulting from their anisotropic shape and abundance of aromatic rings^42^. We therefore hypothesized that we should be able to tune the outcome of the pre-programmed temporal phase changes by the application of a shear force during the annealing process. We refer to this as ‘forging’. Since the chemical changes are determined by the composition of the mixture, it is simple to carry out the pH changes in a highly reproducible manner in a range of sample environments.

**Fig. 3:**
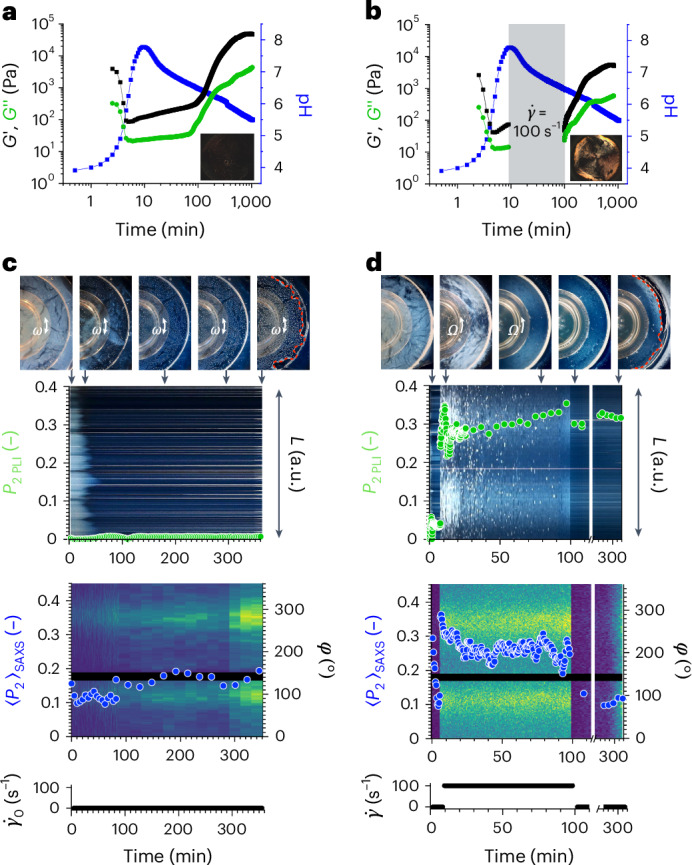
Forging the system by the application of a shear force to induce alignment. **a**, The change in rheology and pH with time for a system containing l,d-2NapFF in the presence of urea, urease and GdL with no unidirectional shear. The inset photograph shows the sample after 16 h using the rheo-optics system. Throughout this figure, the lines and circles are colour coded to the coloured axis titles. **b**, The change in rheology and pH with time for a system containing l,d-2NapFF in the presence of urea, urease and GdL with unidirectional shear starting at 7 min and finishing at 100 min. The inset photograph shows the sample after 16 h using the rheo-optics system. **c**,**d**, Multi-scale analysis for a system with no shear alignment (**c**) and unidirectional shear (**d**) starting at 7 min and finishing at 100 min. Top: photographs of the sample at specific timepoints during the shearing process, indicated with an arrow. The dashed red line in the final image highlights the perimeter of the gel as it dried. Upper middle: time–space diagram of PLI using Herman’s algorithm to detect the presence of the Maltese cross pattern (green). Lower middle: time–space diagram of azimuthally integrated SAXS data with Herman’s orientation parameter (blue). Bottom: the imposed shear rate amplitude (oscillatory shear, **c**) or shear rate (steady shear, **d**). Scalar plot scattering intensity scales can be found in Supplementary Fig. 12. A full description of the calculations to obtain the orientation parameters can be found in Methods. In all cases, [l,d-2NapFF] = 5 mg ml^−1^, [urea] = 0.04 M, [urease] = 0.4 mg ml^−1^ and [GdL] = 14.3 mg ml^−1^. *Ω*, angular velocity of rotating geometry; *ω*, angular frequency; ${\dot{\gamma}}$, applied shear rate; *P*, orientation parameter; *L*, length of arc taken from each PLI frame; *φ*, azimuthal angle. Source data

To forge the system, we apply a shear force to induce alignment. We used a unidirectional shear at specific times during the process using a rheometer. To measure the evolution of *G*′ and *G*′′, oscillatory shear was used during the gel-to-sol transition, followed by a unidirectional shear of 100 s^−1^ starting at 7 min for 93 min. The shear rate was chosen due to optimal observation of alignment in the sol phase (Supplementary Fig. 3a). The time under shear was carefully chosen based on the initial values obtained by rheology and pH (Fig. 2d), to ensure full dispersion of the micellar aggregates in solution as well as to avoid disruption of the final gel. An oscillatory shear was then re-applied to follow the increase of *G*′ and *G*′′ as the sample re-gelled. To observe the effect of shear on the macroscopic scale of the evolving system, a rheo-optics set-up was used; here, alignment can be determined by the observation of a Maltese cross pattern^58,59^. The Maltese cross pattern arises when a structure is preferentially aligned in the flow direction, with the four dark fringes corresponding to the areas where one of the refractive indices of the material under shear coincides with the plane of polarization of the polarizer^58^. If no unidirectional shear is applied, no Maltese cross is observed over the entire process (Fig. 3a, inset and Supplementary Fig. 4c). However, the application of unidirectional shear at 7 min results in the immediate observation of a Maltese cross, showing that the nanotubes are aligning along the direction of shear. If the unidirectional shear is stopped at 100 min, alignment is shown to persist as the pH decreases, with the final system still showing the presence of a Maltese cross (Fig. 3b, inset and Supplementary Fig. 4d). Stopping the shear only after 18 min results in the formation of stiffer materials that exhibit alignment (Supplementary Fig. 5), but with a lower intensity of the Maltese cross (Supplementary Fig. 5c). We hypothesize that the alignment in the sol phase is maintained owing to aromatic inter-fibre interactions^60^, allowing the system to lock in the alignment during re-gelation. If instead the unidirectional shear is maintained up to 300 min, where the apparent p*K*_a_ of the gelator is reached (around 6.0) (ref. ^50^), no re-gelation occurs, and no Maltese cross is observed after 16 h (Supplementary Fig. 7). This shows that the self-assembly process leading to gelation starts to take place earlier than the apparent p*K*_a_ of l,d-2NapFF and careful consideration of timescales is vital in allowing re-gelation of the system while locking in the alignment of the fibres. We note that a similar result can be obtained by the application of unidirectional shear starting at 10 min (Supplementary Fig. 6) when the pH reaches the highest value. However, 7 min was chosen throughout the rest of this study to account for any time loss due to sample loading and measurement start.

To understand how shear alignment affects the nanostructures in solution, rheo-SAXS was performed on the sample. This allowed us to gain information on both the evolution of the mechanical properties of the system and the changes in the underlying self-assembled structures. Alignment of the self-assembled structures in SAXS can be observed by the presence of anisotropy in the two-dimensional (2D) scattering patterns. To expand on this, a novel custom set-up was used to collect polarized light imaging (PLI) data while performing rheo-SAXS, that is, rheo-PLI-SAXS. By doing so, we could further assess the relationship between alignment on the meso- (PLI) and nanoscale (SAXS) under shear. The rheo-SAXS data showed that thin-walled nanotube structures are present throughout the evolution of the system (Supplementary Figs. 9 and 10). When no shear is applied to the system, the PLI data show no Maltese cross across the whole measurement, agreeing with our previous data (Fig. 3a) and indicating no mesoscale alignment (Fig. 3c, green circles). The 2D SAXS pattern shows an initial anisotropy potentially due to sample preparation in the rheometer and squeeze flow to gap. However, the anisotropy immediately drops during measurement, suggesting that no nanoscale orientation is present in the system as it evolves (Fig. 3c, blue circles). A slight increase in nanoscale anisotropy is observed for this sample as it re-gels. This can be ascribed to confinement of the sample with increased bubble formation and drying (Fig. 3c, top, dashed red line). Conversely, a Maltese cross is immediately observed if mono-directional shear is started at 7 min (Fig. 3d, green circles), accompanied by a sudden increase in anisotropy in the 2D SAXS pattern of the system (Fig. 3d, blue circles). A decrease in colour of the Maltese cross can be seen over the shearing window. As the pH of the sample decreases, a slight decrease in radius of the nanotubes is observed (Supplementary Fig. 9c and Supplementary Table 1) and higher interactions between the structures within the system are expected to occur. Such changes can be related to the differences in intensity of the Maltese cross pattern. However, as the sample re-gels, the Maltese cross pattern persists, indicating formation of aligned domains within the final material at the mesoscale level (Fig. 3d, green circles). Interestingly, the nanoscale anisotropy drops once the shear is stopped (Fig. 3d, blue circles). It is likely that, on the nanoscale, entanglements and further aggregations between the self-assembled structures are occurring to allow re-gelation, resulting in relaxation of the anisotropy at this length scale. The one-dimensional scattering patterns of the system do not appear to change greatly between the sheared material and the one with no shear applied, with the presence of thin-walled nanotubes observed in both final materials (Supplementary Figs. 9 and 10 and Supplementary Tables 1 and 2). A slight decrease of about 1.0 nm in radius is only observed for the final gel obtained under shear, which could result from tighter packing of the gelator molecules upon reduction of bubbles during shearing (Supplementary Fig. 9 and Supplementary Table 1). As the SAXS data shown here extend up to 240 nm, we expect that any rupture of the fibres could be observed at this length scale. Hence, our data overall suggest that the shearing process does not break the self-assembled structures under the application of mechanical stimuli.

We can therefore use an external force to influence the outcome of our transient system. A consequence of this is that the mechanical properties of the system are affected by the alignment, with the absolute values of *G*′ and *G*″ being lower as compared with the ones obtained when no unidirectional shear is applied (Fig. 3a,b and Supplementary Fig. 4). These changes are only due to the application of shear, as the structures that underpin the final gel phase are unaffected by the shearing process. Although materials with aligned domains can also be formed directly by gelation under shear^36^, our forging method provides a further level of structural control: here, a variety of gels with different mechanical properties can be obtained from a single starting gel by the application of shear.

**Fig. 4:**
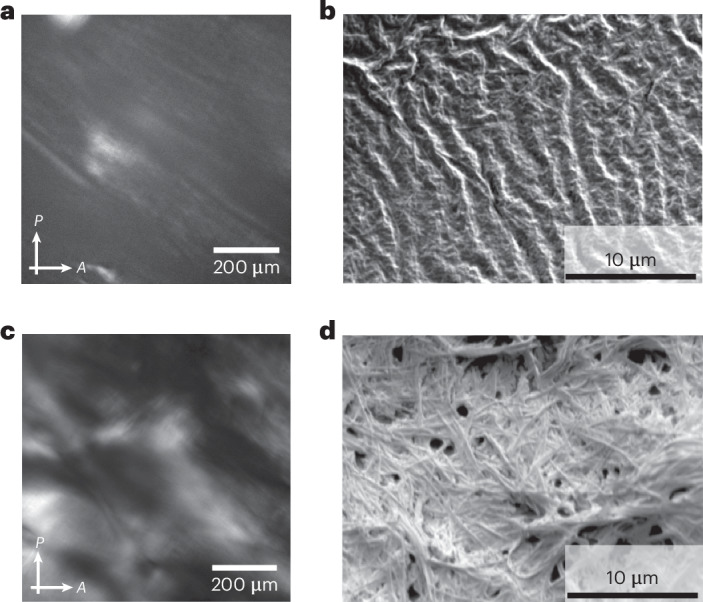
Forging by magnetic alignment within an MRI scanner. **a**,**b**, A cross-polarized optical image (**a**) and SEM image (**b**) for a system containing l,d-2NapFF in the presence of urea, urease and GdL allowed to evolve overnight in an MRI magnet. The vectors labelled P and A are the polarizer and analyzer axes, respectively. **c**,**d**, A cross-polarized optical image (**c**) and SEM image (**d**) for a system containing l,d-2NapFF in the presence of urea, urease and GdL allowed to evolve overnight outside of the magnet. Note that all the samples were kept in the same room with controlled humidity and temperature. In all cases, [l,d-2NapFF] = 5 mg ml^−1^, [urea] = 0.04 M, [urease] = 0.4 mg ml^−1^ and [GdL] = 14.3 mg ml^−1^.

While effective, the application of shear requires external intervention. The use of a magnetic field can also be used to induce alignment for such systems in a non-invasive manner. Previously, our group has successfully aligned self-assembled structures of nanotubes within a magnetic field in an NMR magnet^42^. The behaviour of l,d-2NapFF within a magnetic field has further been studied using a magnetic sample environment for in situ SAXS, showing that the nanotubes can be successfully oriented using low and moderate magnetic fields (Supplementary Fig. 13). NMR has also been previously used to quantify the extent of alignment of self-assembled oligopeptide nanofibres^61^. The ^2^H NMR spectrum of a l,d-2NapFF gel formed in absence of urea/urease/GdL within an NMR tube indicated a lack of magnetic alignment in the gel phase (Supplementary Fig. 14). For the transient system shown here, the NMR approach was not possible as the small size of the NMR tube did not allow homogeneous preparation of the gel incorporating the three triggers, affecting the kinetics. However, we hypothesized that magnetic alignment could be achieved if the sample was allowed to evolve within a larger vessel. Hence, samples of l,d-2NapFF containing the competing pH triggers were prepared inside an magnetic resonance imaging (MRI) scanner with a magnetic field strength of 9.4 T. To test the effect of the magnetic alignment, samples were allowed to form in the MRI scanner. Identical systems were prepared outside the MRI as a control. After being left for 16 h, cross-polarized microscopy and scanning electron microscopy (SEM) of the resulting samples showed uni-directional alignment of the final structures orthogonal to the field, both in the gel state (Fig. 4a) and in the dried state (Fig. 4b). Conversely, samples left outside of the MRI magnet showed random orientation of fibres (Fig. 4c,d). As the rheo-PLI-SAXS data show the tendency of such structures to align on the mesoscale, we hypothesize that the periodicity observed in the dried state (Fig. 4b) could result from longer-range alignment between bundles of smaller fibres, as well as clumping of the fibres during drying.

## Conclusions

Here, we present a method to obtain anisotropic materials by changing the organization of the supramolecular fibres using external stimuli as the sample evolves through pre-programmed phase changes. Material properties can be directed through a forging approach, where external force is used to direct alignment of the supramolecular fibres and impart different mechanical responses. This method allows tunable, temporal control over network and final material properties. Dynamic systems are of wide interest, and here we provide a method for further controlling systems beyond the previous quiescent systems. While we have exemplified this for a single system, many functionalized dipeptides and other gelators form gels under one set of conditions and micellar phases under other conditions and hence we see no reason why this approach is not generalizable. There is a wider contextual interest here too, as many dynamic biological systems adapt in a flowing environment. We also show that our system is versatile, and that anisotropy can be further introduced in the sample by the application of a magnetic field, enabling changes in network in a non-invasive manner. Aligned peptide fibres and nanotubes have applications in directing cell growth^62^. Additionally, aligning fibres and tubes has potential usefulness in optoelectronic applications^63,64^. We envisage that our approach may be used to design adaptive materials for these applications.

## Methods

### Materials

l,d-2NapFF was synthesized as described previously^50^. DMSO (≥99%) was purchased from Fischer Scientific. Urease (U4002-100KU, Jack Beans, 100,000 units g^−1^ solid), urea (ultrapure 99%) and GdL (99%) were purchased from Alfa Aesar. All chemicals were used as received. Deionized water was used throughout all experiments.

### Solution preparation

To prepare the stock solution of l,d-2NapFF, the gelator powder was dissolved in DMSO to reach a concentration of 200 mg ml^−1^. Stock solutions of urea and urease were prepared in H_2_O, at concentrations of 4 M and 0.450 mg ml^−1^, respectively. The concentration of the urease stock solution was determined by taking the mass of the enzyme powder (in mg) dissolved in a known volume of H_2_O. Both urea and urease were highly soluble in water and hence did not require stirring. Stock solutions of GdL were prepared fresh on the day by dissolving GdL in DMSO under stirring to achieve a final concentration of 286 mg ml^−1^.

### Preparation of gels

l,d-2NapFF gels that did not evolve over time were prepared without adding urea, urease and GdL. For these samples, 50 μl of l,d-2NapFF 200 mg ml^−1^ stock solution was diluted with 150 μl of DMSO in a 7 ml Sterilin vial. Then, 1.8 ml of H_2_O was added to this in one aliquot, achieving a final gelator concentration of 5 mg ml^−1^ and a DMSO:H_2_O ratio of 10:90.

l,d-2NapFF gels pre-programmed to undergo gel-to-sol-to-gel transitions were prepared in the presence of urea, urease and GdL. For the preparation of these gels, 50 μl of l,d-2NapFF 200 mg ml^−1^ stock solution was further diluted with 50 μl of DMSO in a 7 ml Sterilin vial. To this, 20 μl of aqueous urea (4 M) and 100 μl of GdL solution (286 mg ml^−1^) were added and mixed by briefly swirling the vial. Finally, 1.78 ml of aqueous urease stock solution (0.45 mg ml^−1^) was added to the mixture in one aliquot to induce gelation. The solvent ratio of DMSO and H_2_O was kept at 10:90 for all samples. The final concentrations of the components were as follows: [l,d-2NapFF] = 5 mg ml^−1^, [urea] = 0.04 M, [urease] = 0.4 mg ml^−1^ and [GdL] = 14.3 mg ml^−1^.

### pH measurements

pH measurements were taken with a HANNA FC200 pH probe using a 6 mm × 10 mm conical tip with ±0.1 accuracy. To monitor the pH changes of the evolving system, the gels were prepared as above immediately before starting the measurement. To keep the temperature constant at 25 °C, the sample was kept in a circulating water bath.

### Rheological measurements

#### Time sweeps

To initially observe the kinetics of the system, time sweeps were performed on an Anton Paar Physica MCR301 rheometer. A cup and vane measuring system was used, set at a measuring distance of 2.1 mm. The test was performed by preparing the sample in a 7 ml Sterilin vial immediately before positioning the vial in the rheometer cup. The tests were performed at a frequency of 10 rad s^−1^ and strain of 0.5% over 16 h.

#### Rheo-optics

All time sweeps were performed on an Anton Paar Physica MCR302 coupled with the shear-induced PLI technique, as previously reported^36,58,59^. For all experiments, a PP25 geometry was used at a measuring distance of 1.0 mm. The gels were prepared directly on the plate by mixing solution of the gelator in DMSO and water (10/90, v/v) in the presence of urea, urease and GdL, and pouring the sample onto the quartz plate. To contain the sample during the gel-to-sol-to-gel transitions, a circular mould with a 32 mm diameter was used. A custom workbook was designed on RheoCompass to apply a constant shear (shear rate of 100 s^−1^) on the system at variable time intervals, while collecting oscillatory shear rheology on the system when no shear was being applied. For all these, oscillatory shear rheology measurements were run at a frequency of 10 rad s^−1^ and strain of 0.5%. To prevent evaporation, wet blue roll was placed around the sample overnight. Cross-polarized images of the materials were taken within the first 20 min and after 16 h to observe the presence of a Maltese cross pattern. A halogen lamp (white light source) was used to illuminate the sample.

Strain sweeps for the gels obtained after the gel-to-sol-to-gel transitions were conducted just after the time sweeps, without lifting the geometry from the surface of the gel. These were performed over the range of 0.1% to 1,000% strain at a constant frequency of 10 rad s^−1^.

#### Rheo-PLI-SAXS

Rheo-PLI-SAXS was performed using a developed custom set-up^65^(Supplementary Fig. 15) adapted from previous custom set-ups used to observe birefringence under flow^66–68^. The set-up is based on a glass parallel plate measuring geometry in a separate motor–transducer configuration. The diameter of the upper plate was 43 mm, with region (i) in the figure being opaque due to the steel shaft of the geometry, and region (ii) being see through (Supplementary Fig. 15a).The custom set-up was adapted on an Anton Paar MCR702 MultiDrive rotational rheometer. Here, the samples were prepared similarly as described in ‘Rheo-optics’ section, using the same custom RheoCompass workbook to apply a constant shear on the system.

The SAXS part consists of a radial incident configuration (R), meaning that the scattering pattern corresponds to the (1–2) plane, where (1) is the velocity direction and (2) is the velocity gradient direction. To assess the flow induced orientation at nanoscale length scale, the scattering patterns were azimuthally integrated within a $q\in \left[\mathrm{1.4,3.4}\right]{\rm{\times }}{10}^{-2}$ Å^−1^ region of interest identified on the basis of radial integration curves. Furthermore, as a quantitative measure of orientation, the azimuthally integrated data were fitted with an element series of even Legendre polynomials to determine the Herman’s orientation parameter as

$${\left\langle {P}_{2}\right\rangle }_{\mathrm{SAXS}}=\frac{\displaystyle{\int }_{-\frac{\pi }{2}}^{\frac{\pi }{2}}1/2\left(3{\rm{co}}{{\rm{s}}}^{2}\varphi -1\right)I\left(\varphi \right)\sin \varphi d\varphi }{\displaystyle{\int }_{-\frac{\pi }{2}}^{\frac{\pi }{2}}I\left(\varphi \right)\sin \varphi d\varphi },$$

where *φ* is the azimuthal angle. We note that, within the integration limits chosen, 〈*P*_2_〉_SAXS_ = 0 signifies random orientation whereas 〈*P*_2_〉_SAXS_ = 1 corresponds to full orientation in the flow direction.

A PLI optical train was positioned perpendicular to the shear plane. The optical train consisted of a cross-polarized set-up operating in transmission mode. A digital single lens reflex (DSLR) Canon 90D camera (Canon) equipped with a 100-mm Canon L-series macro lens was used to perform the PLI flow visualizations. The visualized area corresponds to region (ii) in Supplementary Fig. 15a, which allows one to identify the onset of the Maltese cross pattern. Owing to the extended testing times, images (2,440 × 1,344 px resolution) were acquired at a frequency of 1/5 Hz (every 5 s). Out of each frame, a pixel arc of length *L* was extracted from each frame and was added to a newly created so-called space–time image (Supplementary Fig. 15a). Thus, the *y* axis of the image corresponds to the arc *L* while the *x* axis corresponds to the experimental time *t*. Based on the similitude between the azimuthally integrated scattering data and the greyscale intensity of the space–time diagrams, Herman’s algorithm was used to also quantify the onset of the Maltese cross pattern by replacing *I*(*φ*) with the greyscale intensity of the PLI images, *I*_*g*_ (*α*), where *α* is the angular coordinate along the arc *L*. We note that, in this case, the resulting ${P}_{2{{\mathrm{PLI}}}}$ does not have the same meaning as for SAXS; rather, the same mathematical framework is used to assess the azimuthal uniformity of the flow field as viewed through the PLI:${P}_{2{\mathrm{PLI}}} > 0$ detects the onset of the Maltese cross pattern, that is, when there is unidirectional orientation of optical indicatrixes in the shear direction. We note that, while the notion of optical indicatrix does not specify an associated material lengthscale, it captures contributions from the mesoscale, which complements SAXS orientation material lengthscales on the nanometre scale. Finally, the radius corresponding to the arc of length *L* was *r* = *D*/3 from the centre of the geometry, which corresponds to the radius at which the nominal shear rate was calculated^67^.

### SAXS

SAXS data were collected at the CoSAXS beamline at the diffraction limited 3 GeV storage ring at the MAX IV Laboratory in Sweden. For the rheo-PLI-SAXS measurements, a novel custom set-up was used, as described above. An X-ray beam of 15 keV was used, with the camera length set at 4.7 m to achieve a *q* range of 0.0026–0.3 Å^−1^ (where *q* = 4π/*λ* sin(*θ*), *λ* = 0.8267 Å and 2*θ* is the scattering angle). The data were collected using an EIGER2 4 M hybrid photon counting pixel detector (Dectris AG). The gel samples were prepared directly on the rheometer plate as described above. For all samples, rheo-PLI-SAXS data were collected over 6 h to ensure that gelation had occurred. For the sheared sample, 0.1 s exposures were collected every 30 s within the first 6 min to observe any information on the gel-to-sol transition. As the sample was being sheared, 2 s exposures were recorded every 30 s for 90 min. Finally, 0.1 s frames were collected every 10 min as the sample re-gelled until the end of the run. For the control sample with no shear, shorter exposures were required to avoid X-ray damage. Hence, 0.1 s frames were collected every 30 s within the first 100 min, followed by 0.5 s frames every 20 min until the end of the 6 h. Rheological data and images were collected every 30 s over the course of each run.

SAXS data of l,d-2NapFF within a magnetic field were collected using the electromagnetism set-up of the CoSAXS beamline. The X-ray beam used had an energy of 15 keV, and the sample–detector distance was set at 4.5 m. A GMW 3480 dipole electromagnet magnet with 5-mm-diameter poles was used for the experiments. The poles distance was set at 2 mm distance to achieve a 3 T maximum magnetic field. The samples were measured in 1.5-mm-diameter borosilicate glass capillaries supported by an aluminium frame between the magnetic poles. The magnetic field was swept up from 0 to 3 T in 40 min, holding the sample at 0 T, 1 T and 2 T for 10 min, respectively. Then, the sample was kept at 3 T for 1 h, before lowering the magnetic field down to 0 T. To investigate whether loss of anisotropy occurred immediately below 3 T or over different values, the field was lowered stepwise, holding the sample at 2 T and 1 T for 10 min before reaching 0 T. Then, the sample was held at 0 T for another hour. SAXS data were collected every 5 min with 0.5 s exposure time across the whole magnetic field programme.

### Alignment in the MRI

To align the samples in the MRI, a Bruker 9.4 T MR scanner was used at the Centre for Pre-Clinical Imaging at the University of Liverpool. For these experiments, samples undergoing gel-to-sol-to-gel transitions in the presence of urea, urease and GdL were prepared within the magnet and outside the MRI. All samples were prepared as described above and immediately poured from the vial into a borosilicate glass cell culture dish before gelation. In both cases, six samples were prepared: three were kept sealed and three were left without the lid to allow to dry overnight. The direction of the magnetic field was written on each of the dishes within the magnet. For the MRI samples, the glass dishes were moved into the centre of the magnetic field. The samples prepared outside of the magnet were kept in the same room as the MRI to ensure the samples were subjected to similar environmental conditions. The room was kept at a controlled temperature of 25 °C. After 16 h, the samples were removed from the MRI and directly measured under the microscope.

### SEM

SEM images were collected on a computer-controlled Tescan Clara ultrahigh-resolution scanning electron microscope with a field emission gun electron source (accelerating voltage 0.5–30 keV). The hydrogels were prepared in glass dishes as described in the MRI section and left to dry for a minimum of 24 h. The glass dishes were scored and cut without perturbing the xerogels into ca. 1 cm × 2 cm slides. To prevent charging and obtain better images, the dried hydrogels attached to the glass were sputter coated before imaging using a PolaronSC7640 auto/manual high-resolution sputter coater with a gold–palladium target.

### Optical microscopy under cross-polarized light

Optical microscope images were collected at the Centre for Cell Imaging at the University of Liverpool. The images were recorded using a Zeiss Axio Observer Z.1 (Zeiss) with a 10×/0.3 lens and plane-polarizing filters. An Andor (ANDOR technology) iXon Ultra 897 camera was used to image the samples. All images were acquired using Micro-Manager1.4.15 open-source software (http://www.micro-manager.org/).

### Vitrification of sample and cryo-EM imaging

The sample was vitrified on lacey carbon grid using a Vitrobot Mark IV (Thermo Fisher Scientific). First, the surface of lacey carbon grid was made hydrophilic by glow discharging it in the GloQube (Quorum Technologies). Then, ~3 µl of sample was applied on the glow-discharged grid, and excess sample was blotted away by Whatman filter paper (1001–055) leaving the thin film of sample on grid, which was plunge frozen in liquid ethane. The frozen grid was imaged on a 200 keV cryo-electron microscope (Glacios, Thermo Fisher Scientific) equipped with a cryo-autoloader, field emission gun (XFEG^TM^) electron source and Falcon4 direct electron detector, housed in the University of Virginia Molecular Electron Microscopy Core facility.

## Supplementary information

Supplementary Information Supplementary information, Figs. 1–15 and Tables 1–3.

Supplementary Data 1 Source data for Supplementary Figs. 2–11.

## Source data

Source Data Fig. 2 Rheology data for Fig. 2d and in the main manuscript.

Source Data Fig. 3 Rheology data for Fig. 3a and in the main manuscript.

## Data Availability

All data are available in the main text or Supplementary Information or available on request. This work did not produce any code. Source data are provided with this paper.
