# Supplementary material for: Forging out-of-equilibrium supramolecular gels
Source: Nat Synth. 2024 Sep 6;3(12):1481–9. doi: 10.1038/s44160-024-00623-4 (PMC11628395; doi:10.1038/s44160-024-00623-4)
Supplement: Supplementary file 1 — Supplementary information, Figs. 1–15 and Tables 1–3. [file 44160_2024_623_MOESM1_ESM.pdf]

nature synthesis

Supplementary information https://doi.org/10.1038/s44160-024-00623-4

Forging out-of-equilibrium supramolecular gels

In the format provided by the authors and unedited

Table of Contents

1 Supplementary Results 2
1.1 Cryo-EM 2
1.2 Rheology 3
1.2.1 Preliminary rheology 3
1.2.2 Shear rate tests and viscosity 4
1.2.3 Strain sweeps and SIPLI images 5
1.2.4 Time-sweep rheology 6
1.3 Small angle X-ray scattering 9
1.3.1 Rheo-PLI-SAXS 12
1.3.2 *In situ* SAXS in magnetic sample environment 13
1.4 ^2H NMR spectra of DMSO/H_2O gel 14
2 Supplementary Methods 15
2.1 Rheo-PLI-SAXS 15

1 Supplementary Results

1.1 Cryo-EM

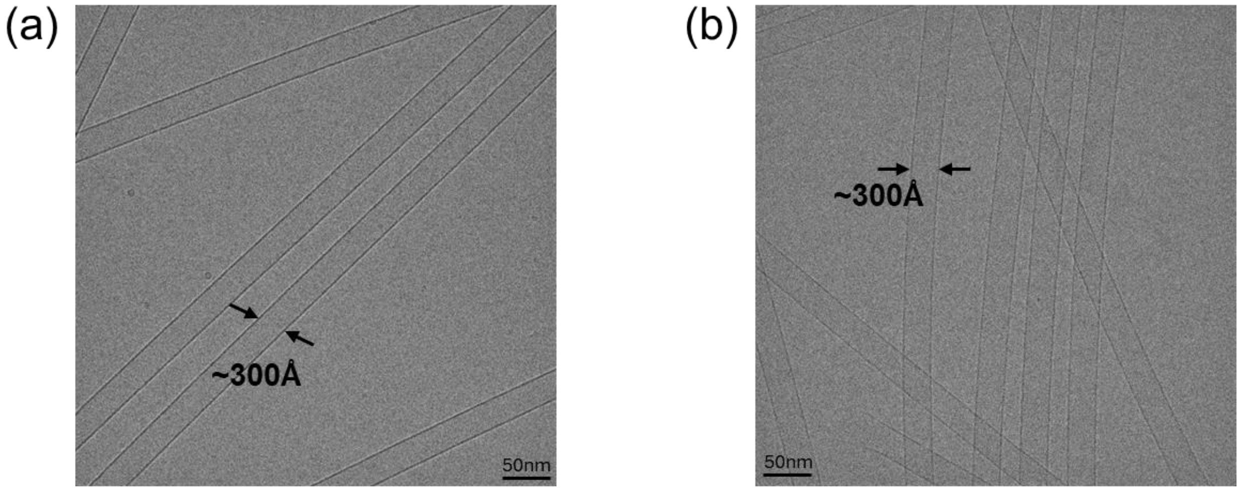

Supplementary Figure 1. Additional cryo-EM images of L,D-2NapFF (scale bar 50 nm), showing the presence of hollow nanotubes within the sample.

1.2 Rheology

1.2.1 Preliminary rheology

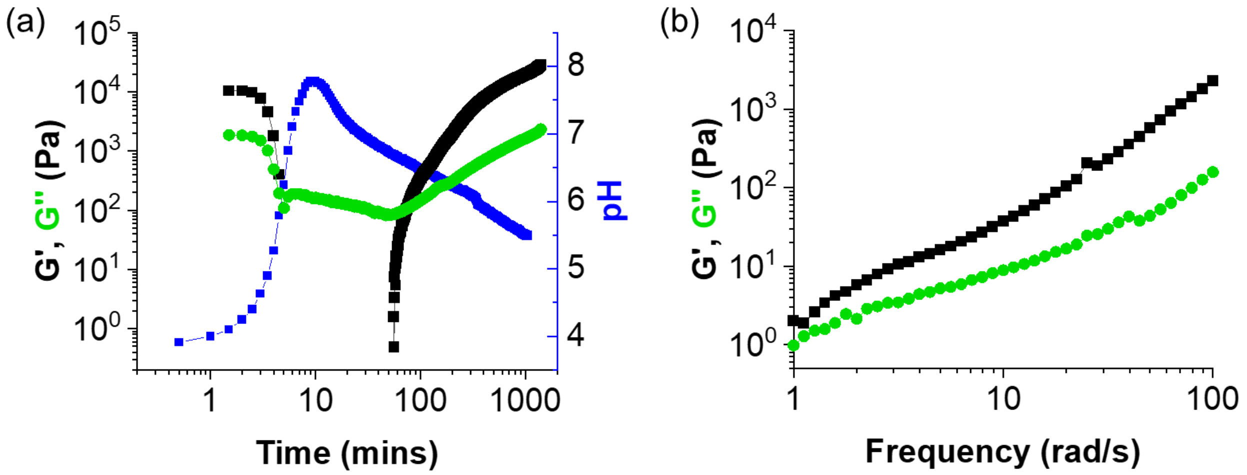

Supplementary Figure 2. (a) Change in rheology and pH with time for a system containing L,D-2NapFF in presence of urea, urease and GdL at ω = 50 rad/s, showing a cross-over in G′ and G″ in the sol phase. (b) Frequency sweep for the solution phase of a system containing L,D-2NapFF in presence of urea, urease and GdL. In all cases, [L,D-2NapFF] = 5 mg/mL, [urea] = 0.04 M, [urease] = 0.4 mg/mL and [GdL] = 14.3 mg/mL.

1.2.2 Shear rate tests and viscosity

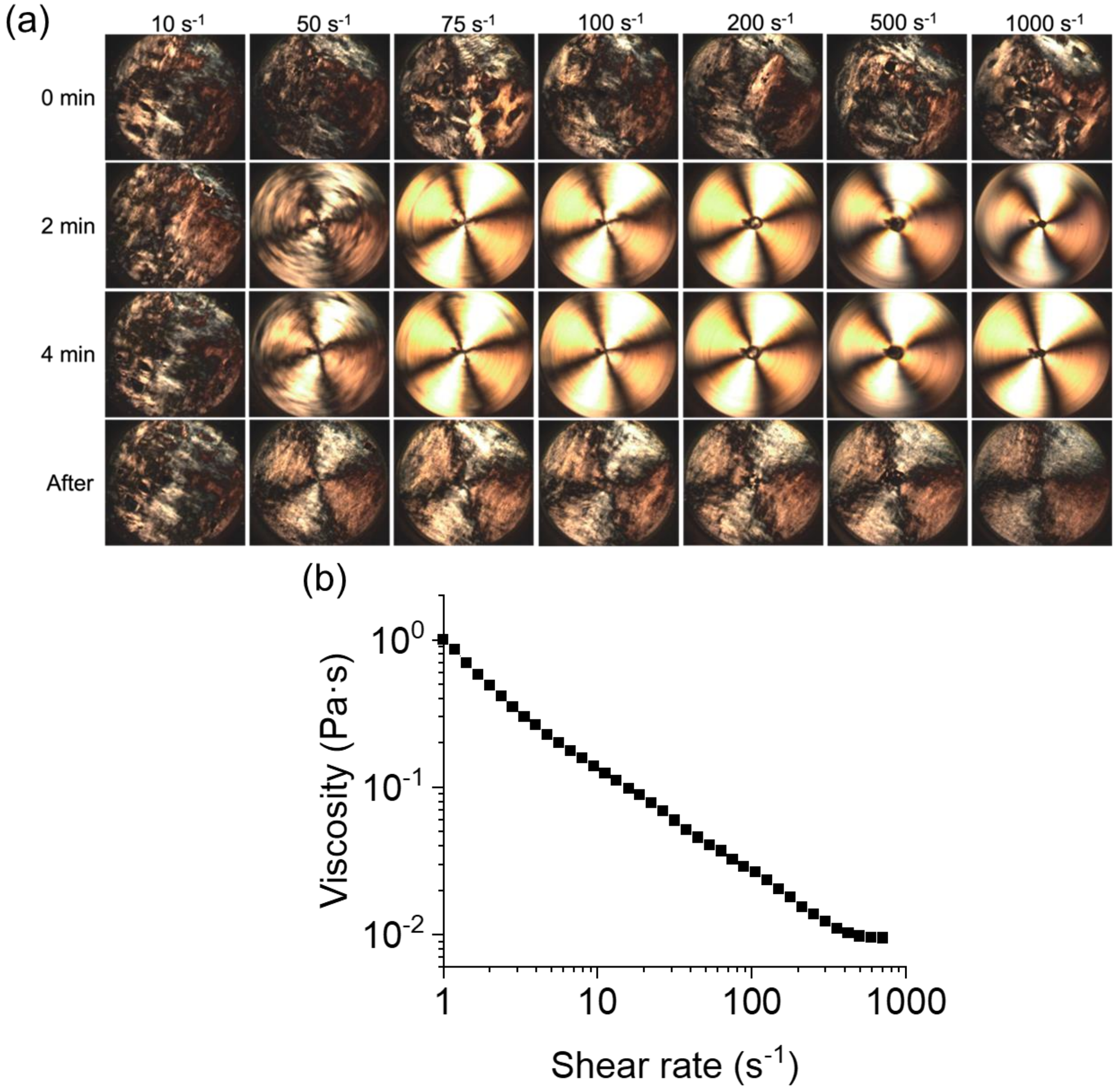

Supplementary Figure 3. (a) SIPLI images of solutions for a system containing L,D-2NapFF in presence of urea, urease and GdL at a range of different constant shear rates (left). Measurements were taken on different samples immediately after the solution phase was formed to best represent the system. All images were taken with the same brightness. (b) Dynamic viscosity measurement for a system containing L,D-2NapFF in presence of urea, urease and GdL in the solution phase.

1.2.3 Strain sweeps and SIPLI images

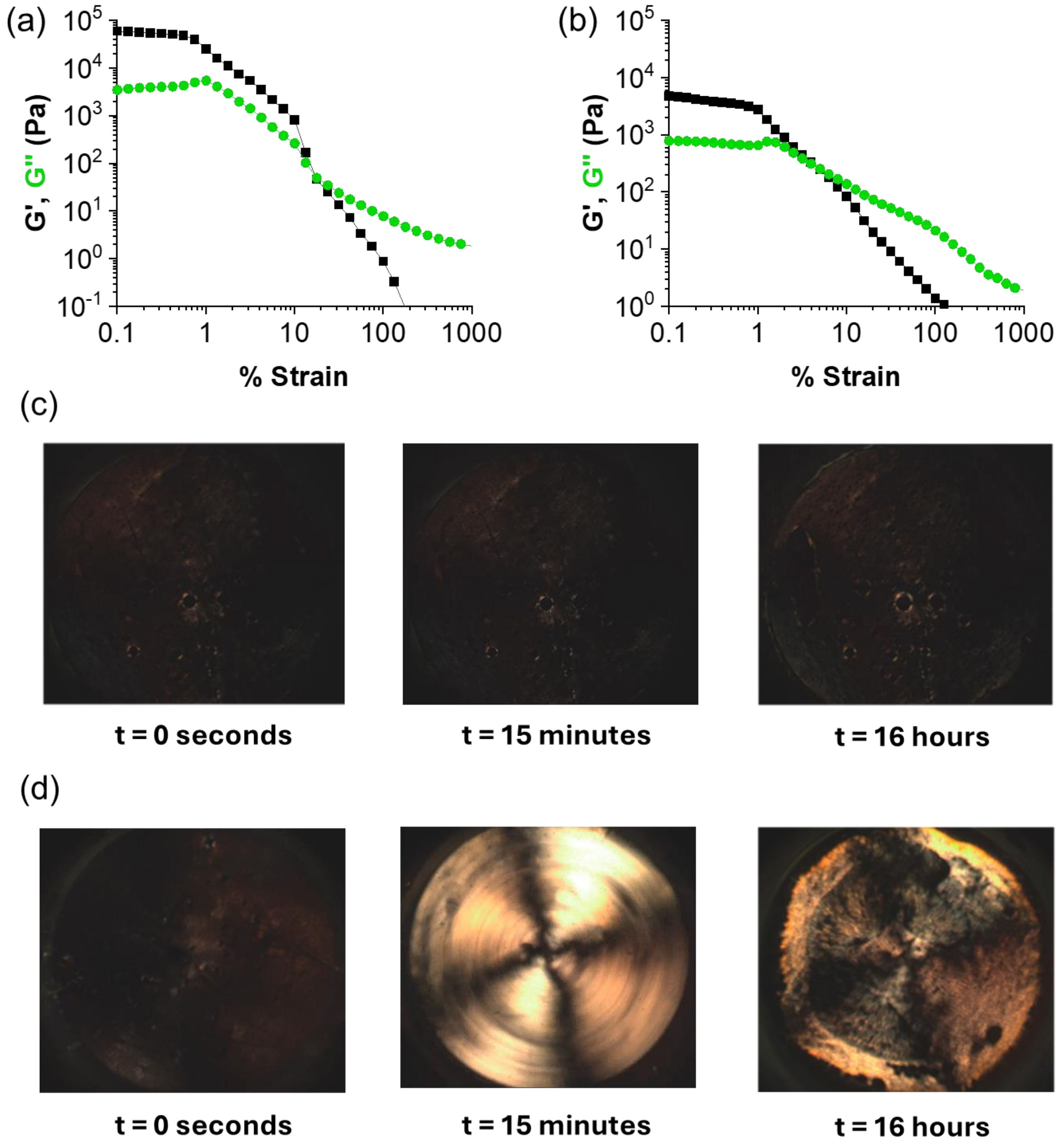

Supplementary Figure 4. Strain sweeps collected after 16 hours for a system containing L,D-2NapFF in presence of urea, urease and GdL with (a) no unidirectional shear and (b) shear applied from 7 minutes to 100 minutes. SIPLI images collected at 0 seconds, 15 minutes and 16 hours a system containing L,D-2NapFF in presence of urea, urease and GdL with (c) no unidirectional shear and (d) shear applied from 7 minutes to 100 minutes In all cases, [L,D-2NapFF] = 5 mg/mL, [urea] = 0.04 M, [urease] = 0.4 mg/mL and [GdL] = 14.3 mg/mL.

1.2.4 Time-sweep rheology

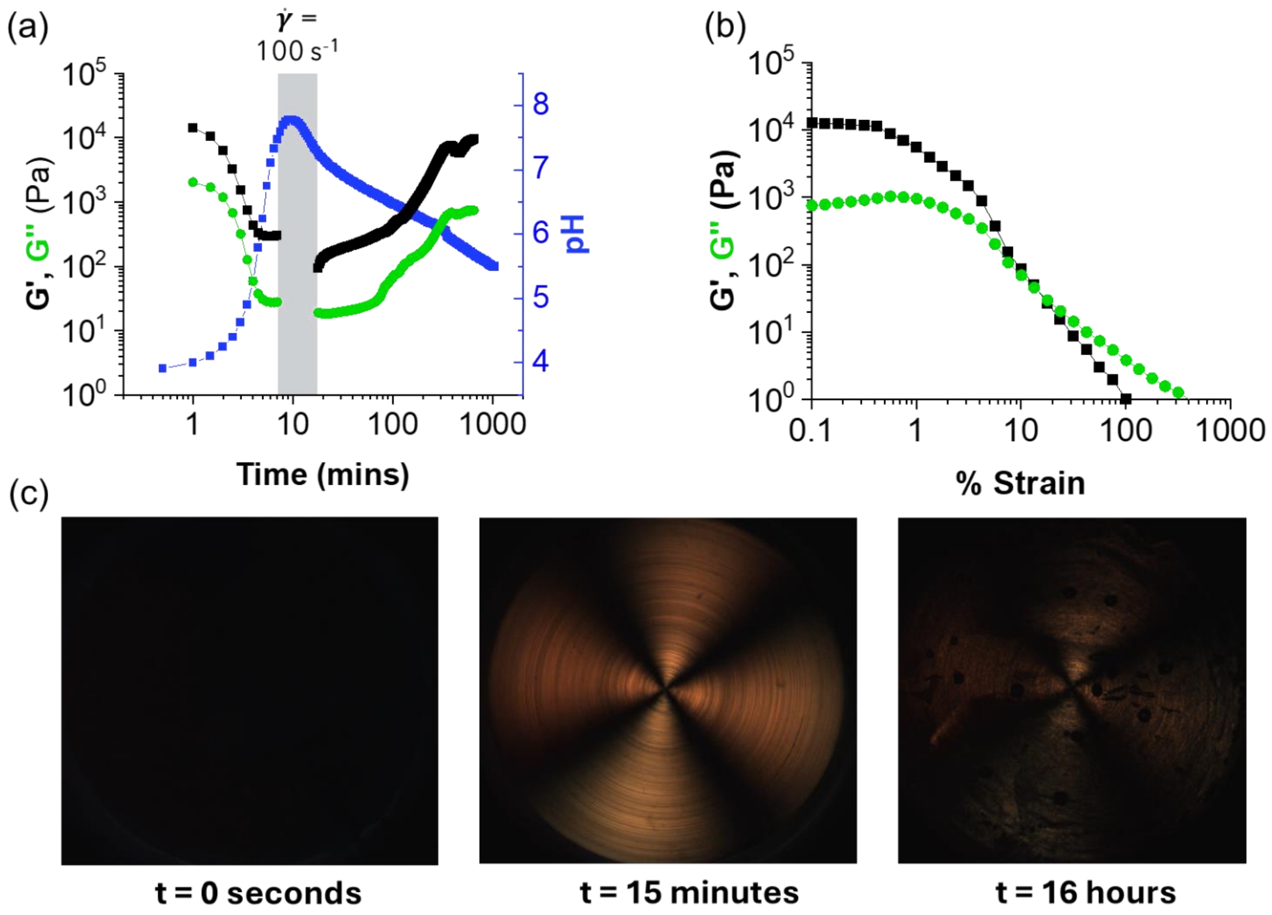

Supplementary Figure 5. (a) Change in rheology and pH with time for a system containing L,D-2NapFF in presence of urea, urease and GdL with unidirectional shear applied from 7 minutes to 18 minutes. (b) Strain sweep collected for the system in (a) after 16 hours. (c) SIPLI images collected at 0 seconds, 15 minutes and 16 hours for a system containing L,D-2NapFF in presence of urea, urease and GdL with shear applied from 7 to 18 minutes. In all cases, [L,D-2NapFF] = 5 mg/mL, [urea] = 0.04 M, [urease] = 0.4 mg/mL and [GdL] = 14.3 mg/mL.

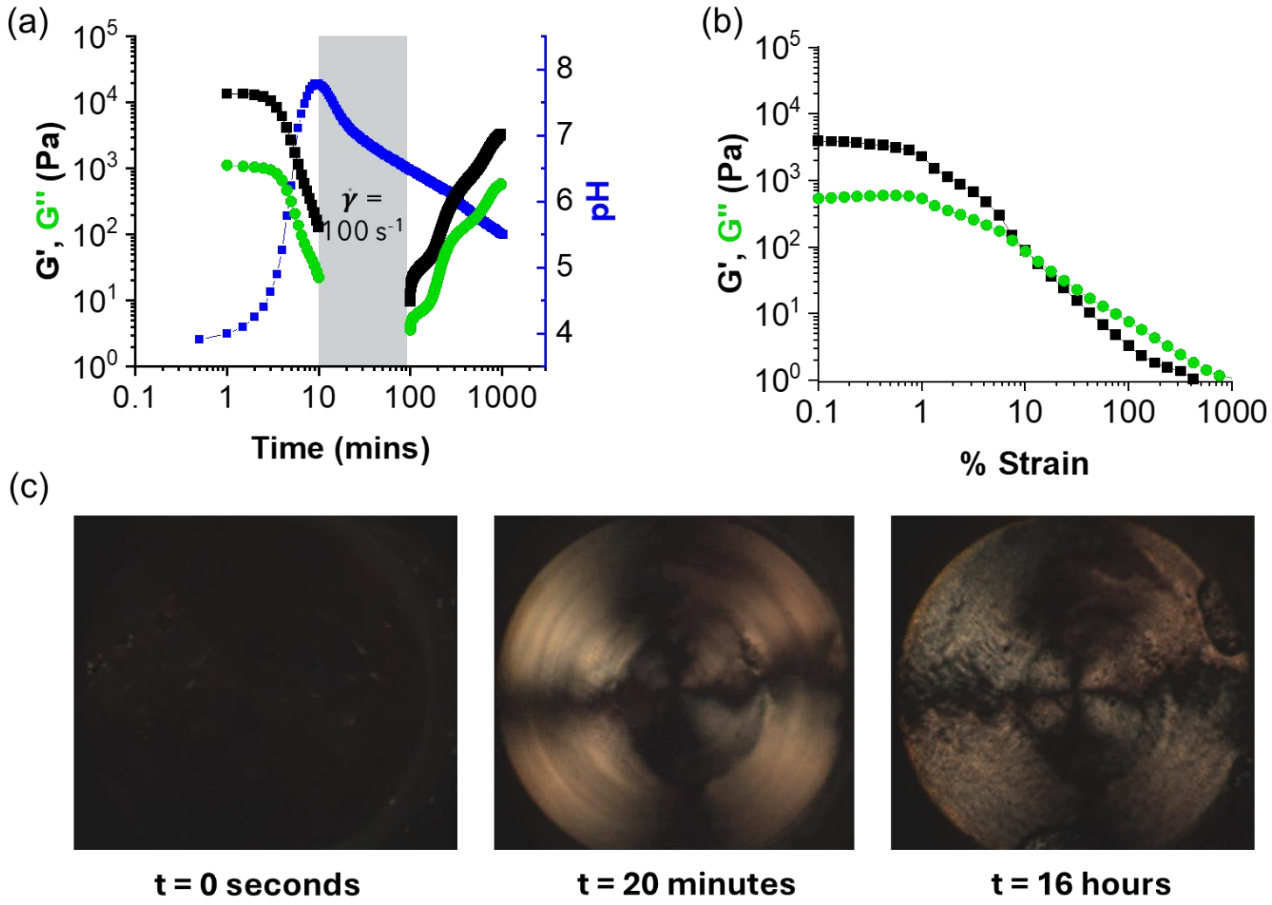

Supplementary Figure 6. (a) Change in rheology and pH with time for a system containing L,D-2NapFF in presence of urea, urease and GdL with unidirectional shear applied from 10 minutes to 100 minutes. (b) Strain sweep collected for the system in (a) after 16 hours. (c) SIPLI images collected at 0 seconds, 20 minutes and 16 hours for a system containing L,D-2NapFF in presence of urea, urease and GdL with shear applied from 10 to 100 minutes. In all cases, [L,D-2NapFF] = 5 mg/mL, [urea] = 0.04 M, [urease] = 0.4 mg/mL and [GdL] = 14.3 mg/mL.

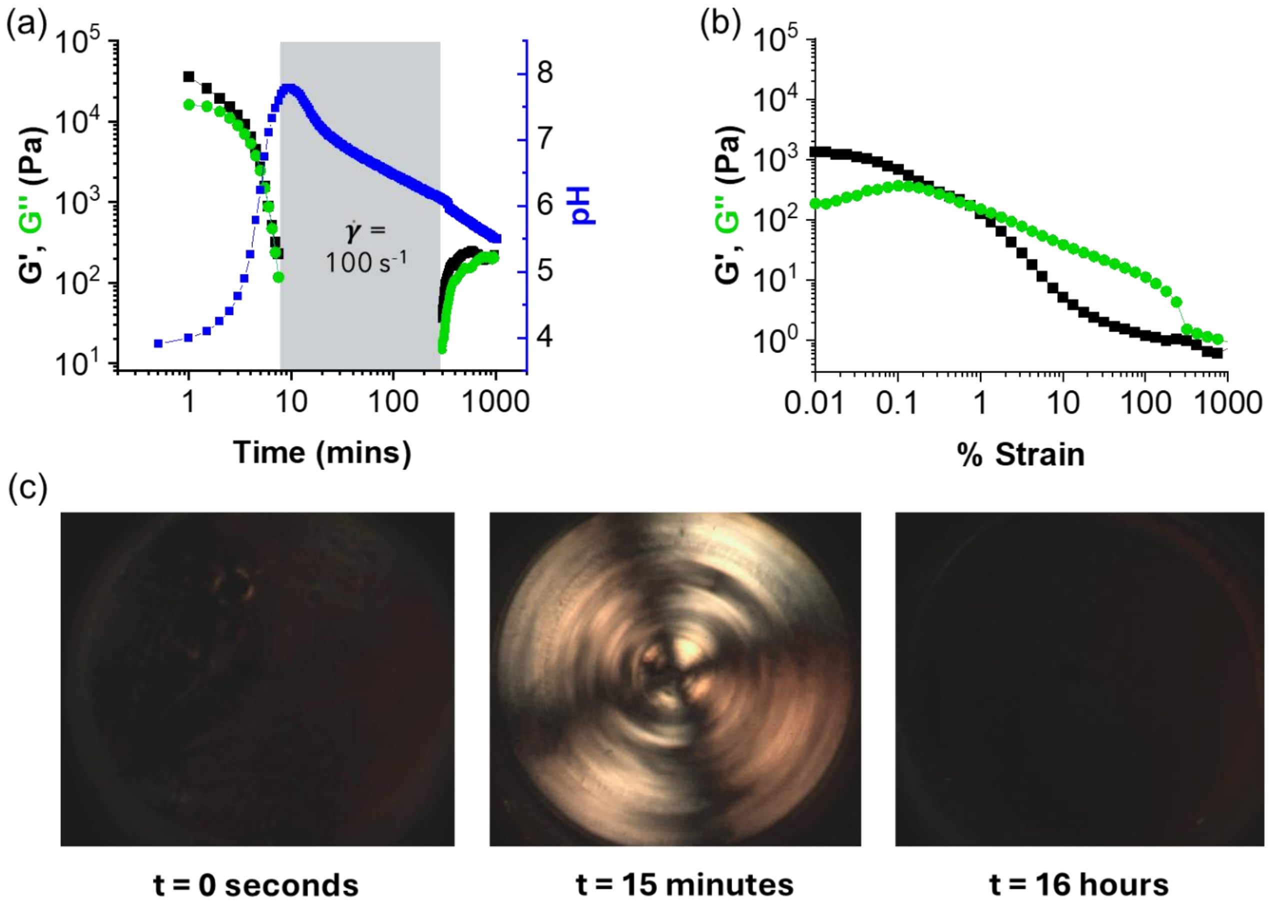

Supplementary Figure 7. (a) Change in rheology and pH with time for a system containing L,D-2NapFF in presence of urea, urease and GdL with unidirectional shear applied from 7 minutes to 300 minutes. (b) Strain sweep collected for the system in (a) after 16 hours. (c) SIPLI images collected at 0 seconds, 15 minutes and 16 hours for a system containing L,D-2NapFF in presence of urea, urease and GdL with shear applied from 7 to 300 minutes. In all cases, [L,D-2NapFF] = 5 mg/mL, [urea] = 0.04 M, [urease] = 0.4 mg/mL and [GdL] = 14.3 mg/mL.

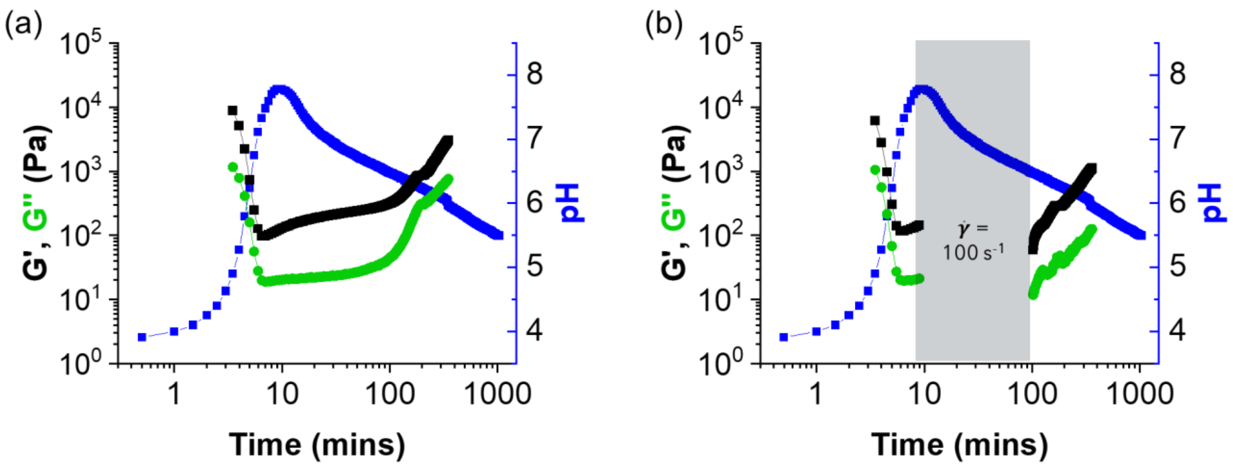

Supplementary Figure 8. Time sweeps for a system containing L,D-2NapFF in presence of urea, urease and GdL with (a) no unidirectional shear and (b) shear applied from 7 minutes to 100 minutes during SAXS collection in the Rheo-PLI-SAXS set-up. In all cases, [L,D-2NapFF] = 5 mg/mL, [urea] = 0.04 M, [urease] = 0.4 mg/mL and [GdL] = 14.3 mg/mL.

1.3 Small angle X-ray scattering

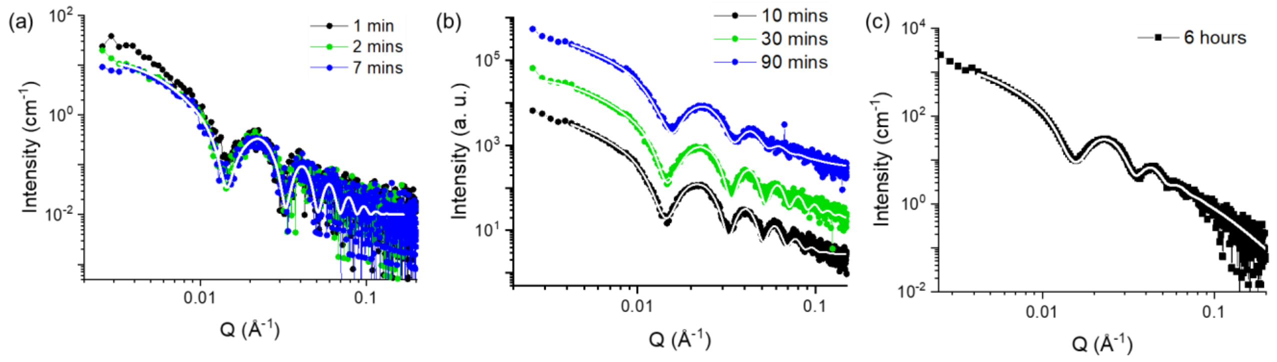

Supplementary Figure 9. Small angle X-ray scattering data of a system containing L,D-2NapFF in presence of urea, urease and GdL with shear applied from 7 minutes to 100 minutes. (a) Data obtained at 1 minute (black data), 2 minutes (green data) and 7 minutes (blue data) under oscillatory shear; (b) data obtained at 10 minutes (black data), 30 minutes (green data) and 90 minutes (blue data) under unidirectional shear; (c) data obtained after 6 hours under oscillatory shear. In all cases, the white line indicates the model fit obtained using Sasview (Supplementary Table 1).

Supplementary Table 1. Fitting parameters for SAXS data in Supplementary Figure 9. All the data was fit to a hollow cylinder model. Values that were manually added and fixed have been labelled with *.

	**(L,D)-2NapFF shear-aligned**									
	**1 – 7.5 mins**		**10 mins**		**30 mins**		**100 mins**		**6 hours**	
	**Value**	**Error**	**Value**	**Error**	**Value**	**Error**	**Value**	**Error**	**Value**	**Error**
**Scale**	0.00025*	/	0.12	0.0017	0.11	0.002	0.11	0.002	0.036	0.0003
**Background (cm^{-1})**	0.008*	/	1.36*	/	1.4*	/	1.89*	/	0.045*	/
**Radius (Å)**	155	0.5551	160.7	0.19	155.94	0.22	147.13	0.27	146	0.13
**Thickness (Å)**	25*	/	19.4	0.28	17.66	0.35	18	0.33	20.63	0.22
**Length (Å)**	1250*	/	1254	17.1	1059.2	16.464	1094.2	21.765	1093	6.99
**Radius polydispersity**	/	/	0.05*	/	0.05*	/	0.1*	/	0.12*	/
χ^2	2.9313		5.80		6.83		11.58		6.96	

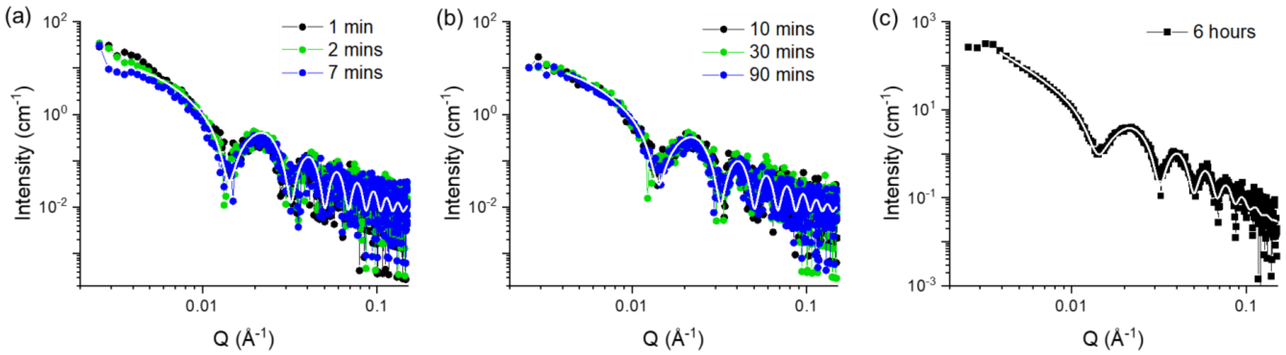

Supplementary Figure 10. Small angle X-ray scattering data of a system containing L,D-2NapFF in presence of urea, urease and GdL with no unidirectional shear applied (a) Data obtained at 1 minute (black data), 2 minutes (green data) and 7 minutes (blue data) under oscillatory shear; (b) data obtained at 10 minutes (black data), 30 minutes (green data) and 90 minutes (blue data) under oscillatory shear; (c) data obtained after 6 hours under oscillatory shear. In all cases, the white line indicates model fit obtained using Sasview (Supplementary Table 2).

Supplementary Table S2. Fitting parameters for SAXS data in Supplementary Figure 10. The data was fit either to a hollow cylinder model (A) or a combination of hollow cylinder (A) and a power law (B). Values that were manually added and fixed have been labelled with *.

	(L,D)-2NapFF no shear					
	1 – 7.5 mins		10 – 30 mins		6 hours	
	Value	Error	Value	Error	Value	Error
Scale	3×10^{-4}*		2.44×10^{-4}	1.47×10^{-5}	1	
Background (cm^{-1})	0.008*		0.008*		0.02	
A_scale	/	/	/	/	2.66×10^{-3}	4.65×10^{-5}
A_radius (Å)	160*	/	160*	/	159.32	0.35
A_thickness (Å)	25*	/	25*	/	27.34	0.56
A_length (Å)	1000*	/	1000*	/	1031.8	21.55
B_scale	/	/	/	/	1.78×10^{-08}	4.56×10^{-09}
B_power	/	/	/	/	4.05	0.05
χ^2	3.814		3.6		8.48	

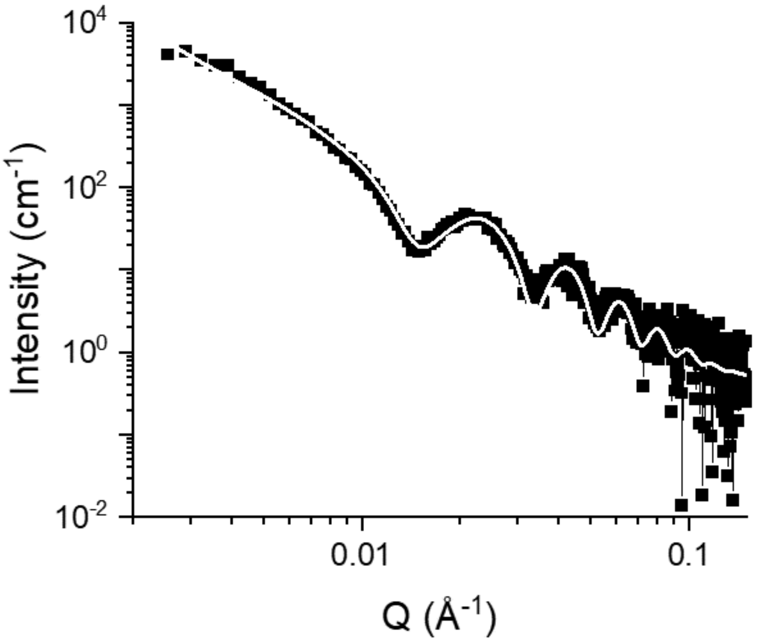

Supplementary Figure 11. Small angle X-ray scattering data of a L,D-2NapFF gel in absence urea and GdL. The gel was formed in presence of urease (0.4 mg/mL) to allow comparison with the evolving systems. The white line indicates model fit through Sasview (Supplementary Table 3).

Supplementary Table 3. Fitting parameters for SAXS data in Supplementary Figure 11. The data was fit to a hollow cylinder model (A) combined with a power law model (B). Values that were manually added and fixed have been labelled with *.

	(L,D)-2NapFF static gel	
	**Value**	**Error**
**Scale**	1	
**Background (cm^{-1})**	0.5*	
**A_scale**	2.44×10^{-2}	6.63×10^{-4}
**A_radius (Å)**	147	0.66
**A_thickness (Å)**	34.2	1.04
**A_length (Å)**	1000*	/
**B_scale**	1.89×10^{-5}	4.74×10^{-6}
**B_power**	3.22	0.045
χ^2	7.03	

1.3.1 Rheo-PLI-SAXS

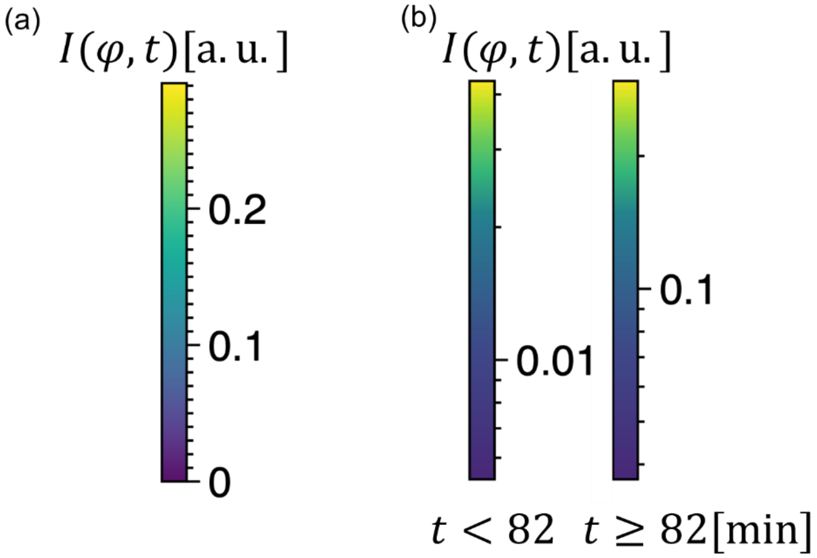

Supplementary Figure 12. Scattering intensity corresponding to the azimuthal integration scalar plots in (a) Figure 3c and (b) Figure 3d in main text.

1.3.2 *In situ* SAXS in magnetic sample environment

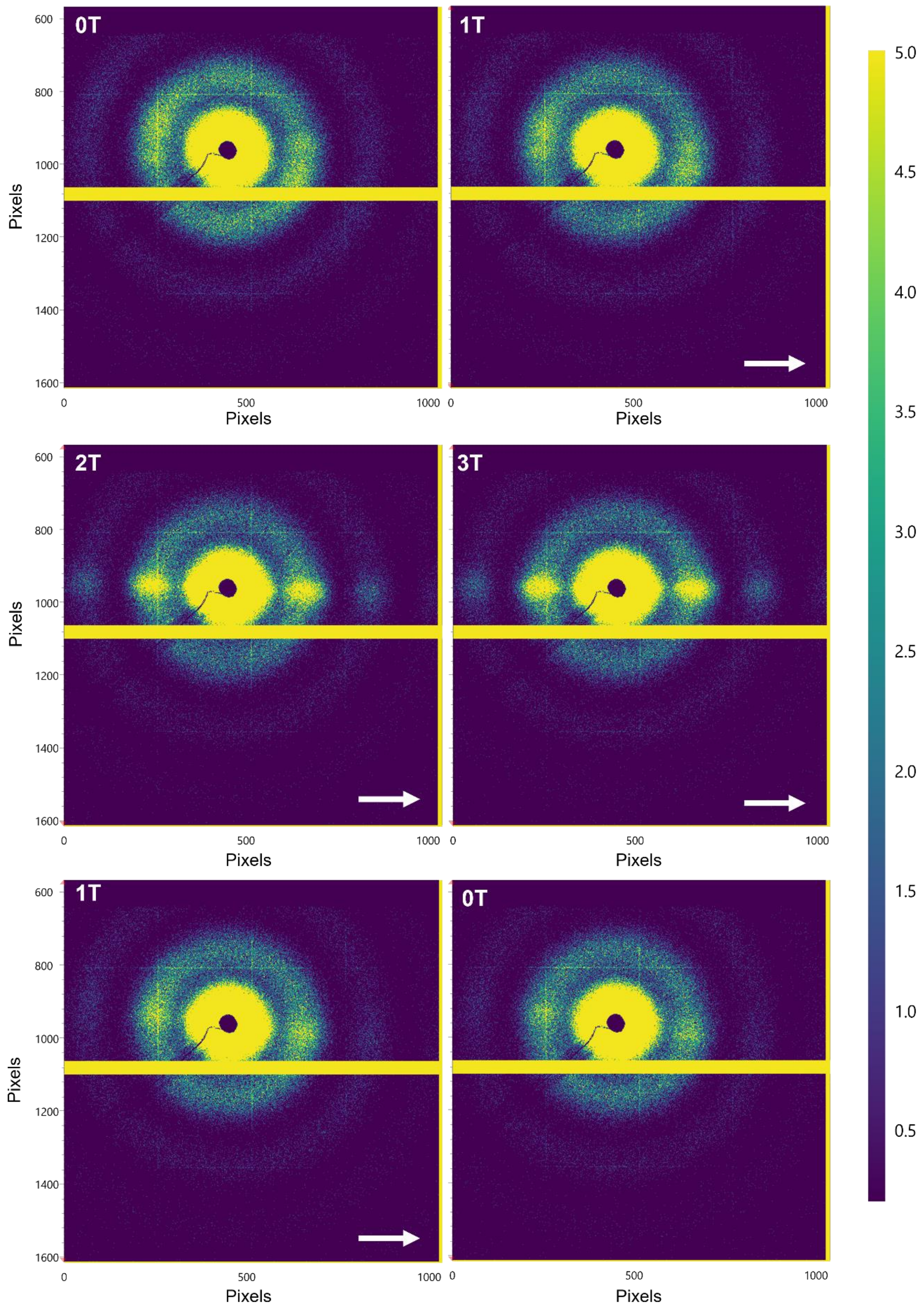

Supplementary Figure 13. Scattering patterns of a 10 mg/mL solution of LD-2NapFF at different strength of applied horizontal magnetic field (white arrow), from 0T to 3T and after relaxation back to 0T. Anisotropy of the scattering pattern in the horizontal field direction at 2T and 3T is indicative of magnetically induced orientation.

1.4 ^{2}H NMR spectra of DMSO/H_2O gel

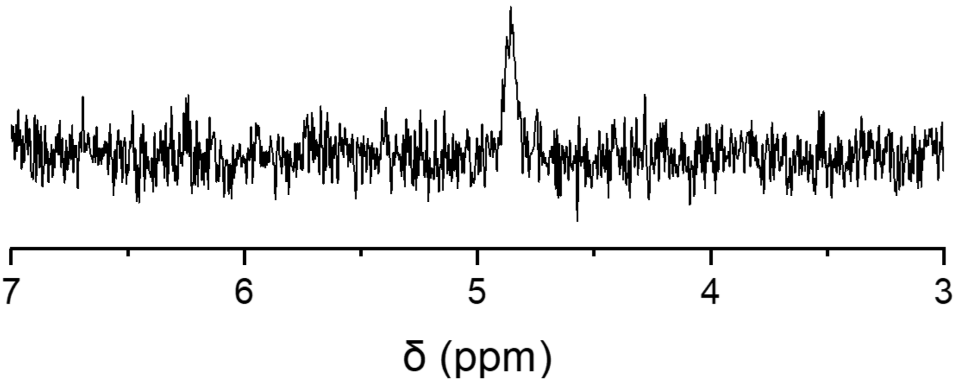

Supplementary Figure 14. ^{2}H NMR spectra of a L,D-2NapFF DMSO/H_2O gel (10/90, v/v) formed in the presence of a magnetic field of 9.4T within an NMR tube. No splitting of the peak can be observed, indicating lack of magnetic alignment.

2 Supplementary Methods

2.1 Rheo-PLI-SAXS

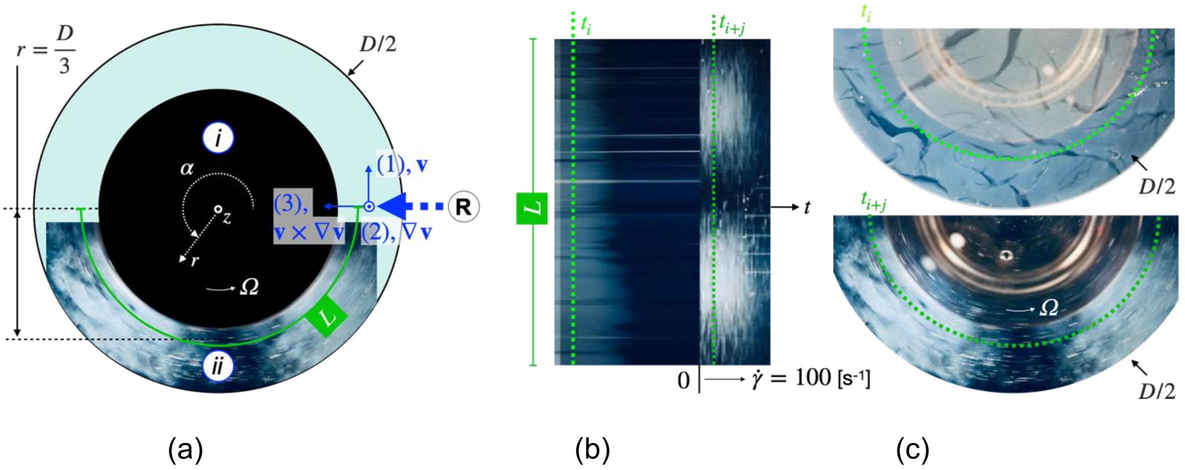

Supplementary Figure 15. (a) Illustration of the rheo-PLI-SAXS setup. (b) Example of space-time diagram. (c) Examples of PLI visualizations acquired during the experiments at rest and during shear, the latter showing a Maltese-cross pattern, and their correspondence to the space-time diagram in (b).
